# TOOLS TO SUPPORT NEEDS ASSESSMENTS ON DEMENTIA, COGNITIVE HEALTH, AND CAREGIVING

**DOI:** 10.1093/geroni/igac059.665

**Published:** 2022-12-20

**Authors:** John Shean, Talyah Sands, Priya Shah, Kelsey Donnellan

**Affiliations:** Alzheimer's Association, Chicago, Illinois, United States; Association of State and Territorial Health Officials, Arlington, Virginia, United States; Association of State and Territorial Health Officials, Arlington, Virginia, United States; Assoc. of State and Territorial Health Officials (ASTHO), Arlington, Virginia, United States

## Abstract

Dementia, cognitive health, and caregiving are not always included in community health needs assessments, leading to their absence in public health improvement and strategic plans, and subsequent lack of action and funding to address these needs. In response, the Alzheimer’s Association and the Association of State and Territorial Health Officials published the Needs Assessment Toolkit for Dementia, Cognitive Health, and Caregiving. Designed for state, local, and territorial public health and aging officials, this toolkit helps communities identify unmet needs of older adults, scale up existing community strengths, and promote healthy aging. The toolkit embeds health equity as a cornerstone to assessing community health. It offers approaches to operationalize equity in the assessment process by including wide representation of people from different racial and ethnic backgrounds, geographic locations, and levels of socioeconomic status and educational attainment. The toolkit’s five steps are modular in design and allow jurisdictions to enter the process at any stage. It considers alignment with jurisdiction-wide plans including Alzheimer’s plans, health improvement plans, aging plans, and the Healthy Brain Initiative Road Map, helping public health agencies advance attention to Alzheimer’s through their networks. Developed through an iterative process, expert input from public health and aging practitioners guided the development and organization. Over 1,600 comments were reconciled to ensure usability among stakeholders, elevate equity to an essential component throughout the assessment process, and add value to allied strategic plans. This presentation complements Learn, Plan, Do: Public Health Planning to Address Dementia and Caregiving, abstract ID 1229181.

